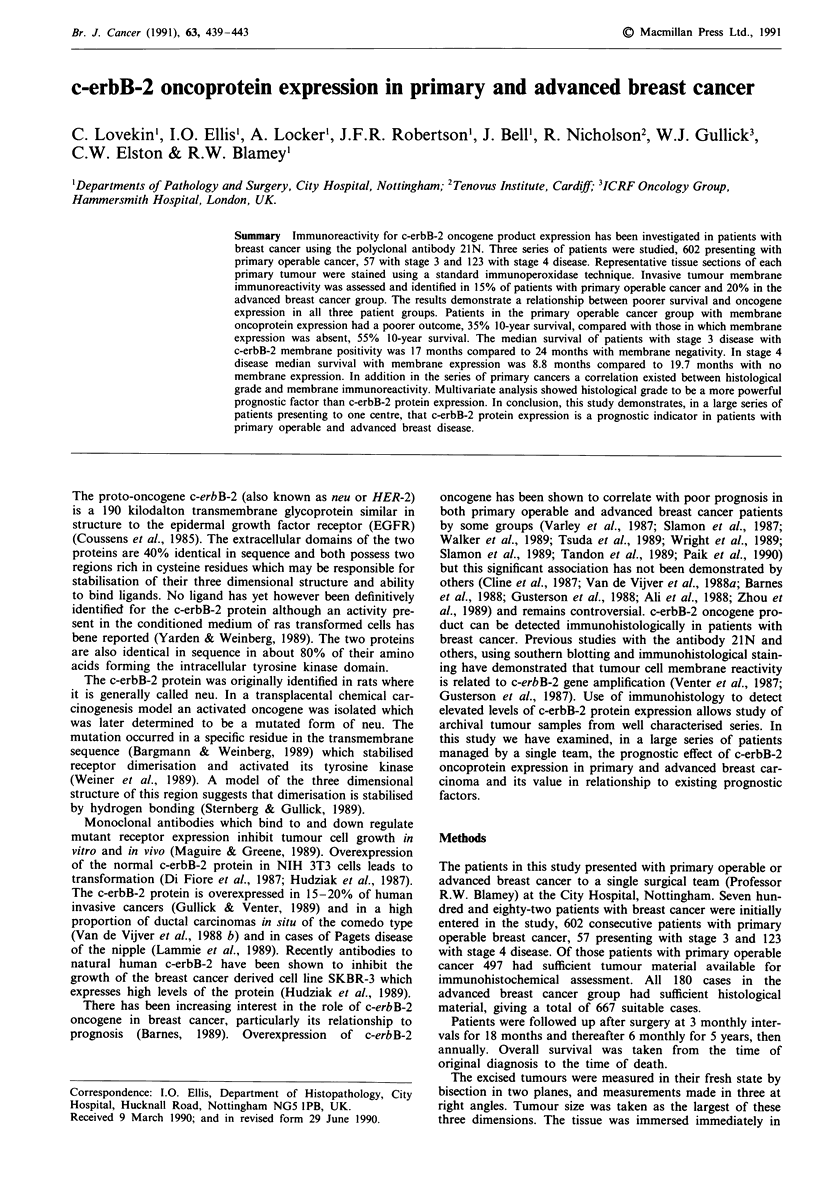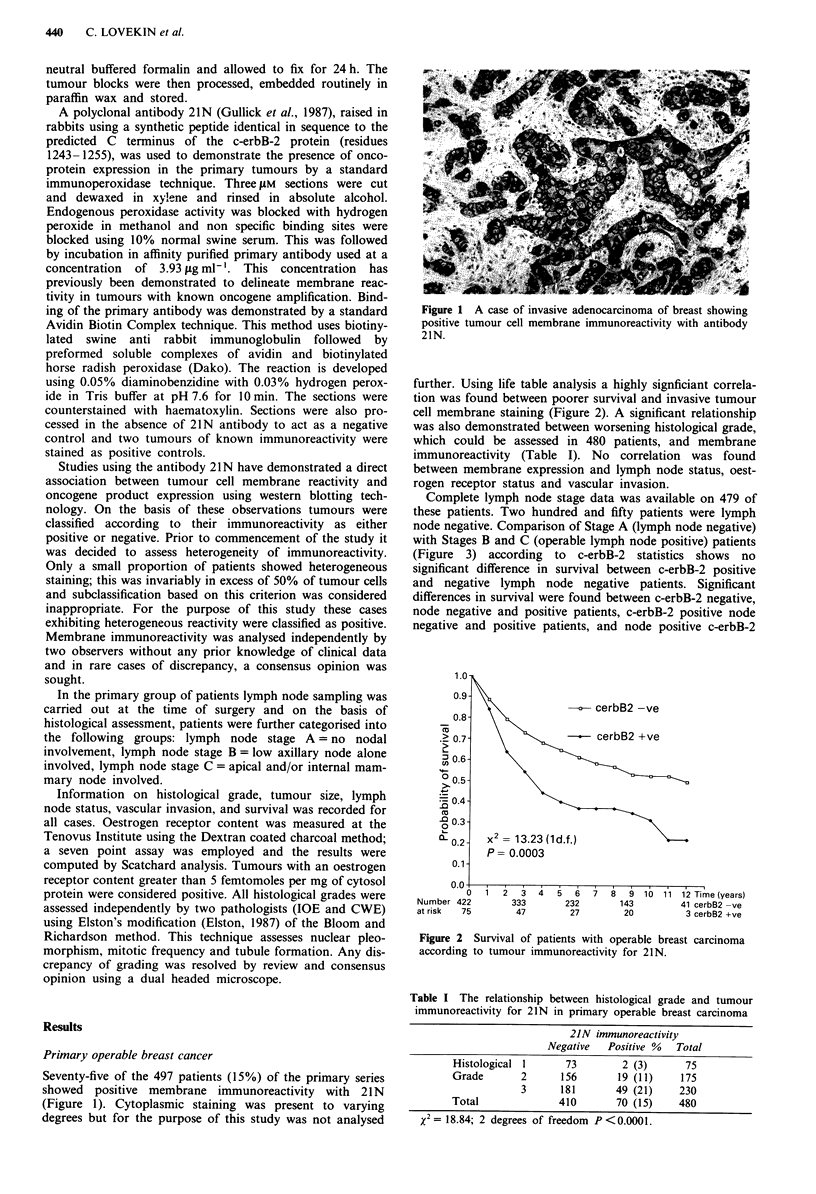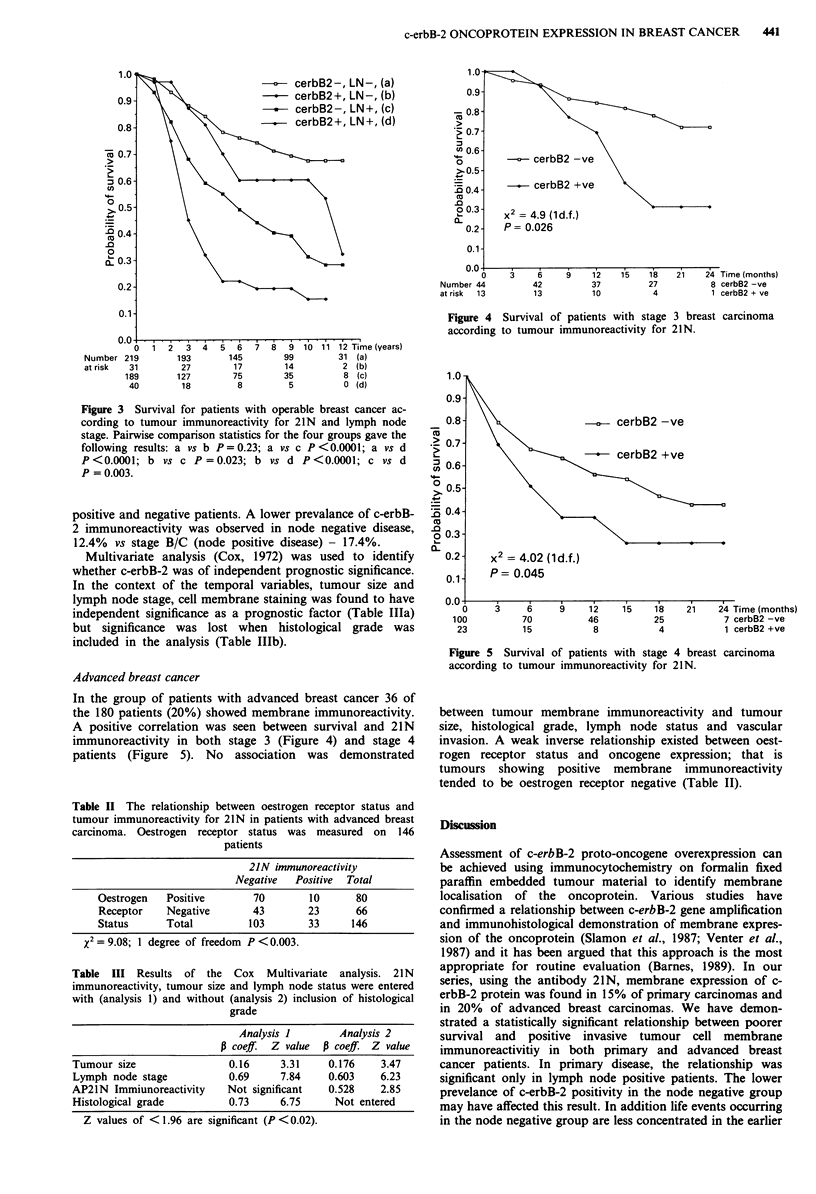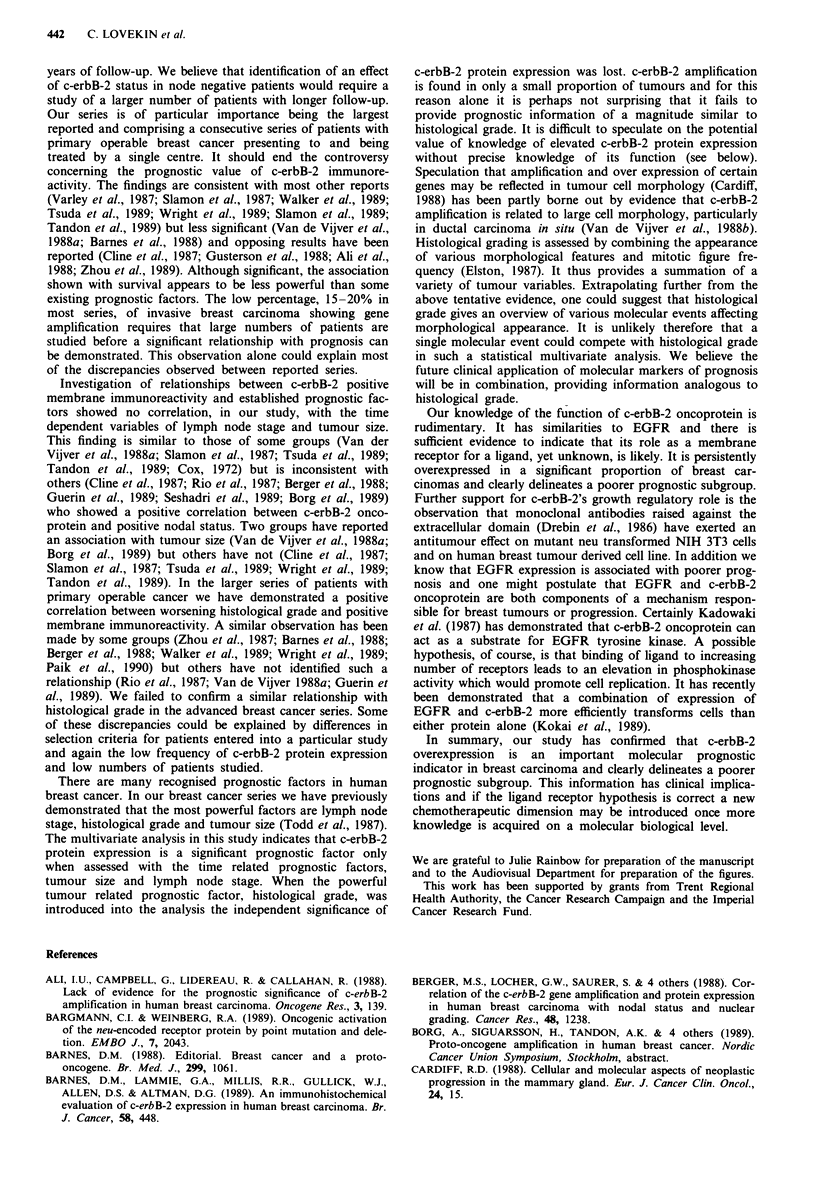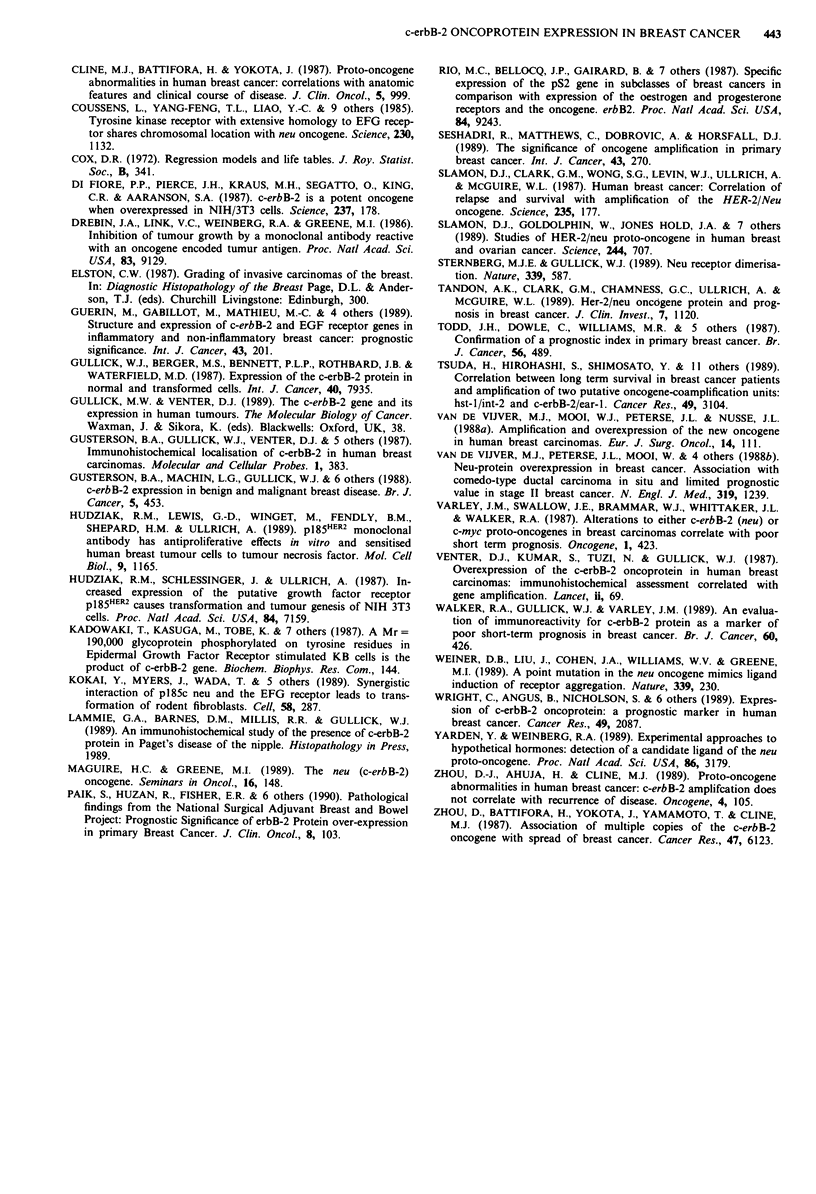# c-erbB-2 oncoprotein expression in primary and advanced breast cancer.

**DOI:** 10.1038/bjc.1991.101

**Published:** 1991-03

**Authors:** C. Lovekin, I. O. Ellis, A. Locker, J. F. Robertson, J. Bell, R. Nicholson, W. J. Gullick, C. W. Elston, R. W. Blamey

**Affiliations:** Department of Pathology, City Hospital, Nottingham, UK.

## Abstract

**Images:**


Br. J. Cancer (1991), 63, 439–443 © Macmillan Press Ltd., 1991

### c-erbB-2 oncoprotein expression in primary and advanced breast cancer

C. Lovekin[1], I.O. Ellis[1], A. Locker[1], J.F.R. Robertson[1], J. Bell[1], R. Nicholson[2], W.J. Gullick[3], C.W. Elston & R.W. Blamey[1]

[1]*Departments of Pathology and Surgery, City Hospital, Nottingham;* [2]*Tenovus Institute, Cardiff;* [3]*ICRF Oncology Group, Hammersmith Hospital, London, UK.*

**Summary** Immunoreactivity for c-erbB-2 oncogene product expression has been investigated in patients with breast cancer using the polyclonal antibody 21N. Three series of patients were studied, 602 presenting with primary operable cancer, 57 with stage 3 and 123 with stage 4 disease. Representative tissue sections of each primary tumour were stained using a standard immunoperoxidase technique. Invasive tumour membrane immunoreactivity was assessed and identified in 15% of patients with primary operable cancer and 20% in the advanced breast cancer group. The results demonstrate a relationship between poorer survival and oncogene expression in all three patient groups. Patients in the primary operable cancer group with membrane oncoprotein expression had a poorer outcome, 35% 10-year survival, compared with those in which membrane expression was absent, 55% 10-year survival. The median survival of patients with stage 3 disease with c-erbB-2 membrane positivity was 17 months compared to 24 months with membrane negativity. In stage 4 disease median survival with membrane expression was 8.8 months compared to 19.7 months with no membrane expression. In addition in the series of primary cancers a correlation existed between histological grade and membrane immunoreactivity. Multivariate analysis showed histological grade to be a more powerful prognostic factor than c-erbB-2 protein expression. In conclusion, this study demonstrates, in a large series of patients presenting to one centre, that c-erbB-2 protein expression is a prognostic indicator in patients with primary operable and advanced breast disease.

The proto-oncogene c-*erb*B-2 (also known as *neu* or *HER*-2) is a 190 kilodalton transmembrane glycoprotein similar in structure to the epidermal growth factor receptor (EGFR) (Coussens *et al.*, 1985). The extracellular domains of the two proteins are 40% identical in sequence and both possess two regions rich in cysteine residues which may be responsible for stabilisation of their three dimensional structure and ability to bind ligands. No ligand has yet however been definitively identified for the c-erbB-2 protein although an activity present in the conditioned medium of ras transformed cells has bene reported (Yarden & Weinberg, 1989). The two proteins are also identical in sequence in about 80% of their amino acids forming the intracellular tyrosine kinase domain.

The c-erbB-2 protein was originally identified in rats where it is generally called neu. In a transplacental chemical carcinogenesis model an activated oncogene was isolated which was later determined to be a mutated form of neu. The mutation occurred in a specific residue in the transmembrane sequence (Bargmann & Weinberg, 1989) which stabilised receptor dimerisation and activated its tyrosine kinase (Weiner *et al.*, 1989). A model of the three dimensional structure of this region suggests that dimerisation is stabilised by hydrogen bonding (Sternberg & Gullick, 1989).

Monoclonal antibodies which bind to and down regulate mutant receptor expression inhibit tumour cell growth *in vitro* and *in vivo* (Maguire & Greene, 1989). Overexpression of the normal c-erbB-2 protein in NIH 3T3 cells leads to transformation (Di Fiore *et al.*, 1987; Hudziak *et al.*, 1987). The c-erbB-2 protein is overexpressed in 15–20% of human invasive cancers (Gullick & Venter, 1989) and in a high proportion of ductal carcinomas *in situ* of the comedo type (Van de Vijver *et al.*, 1988 *b*) and in cases of Pagets disease of the nipple (Lammie *et al.*, 1989). Recently antibodies to natural human c-erbB-2 have been shown to inhibit the growth of the breast cancer derived cell line SKBR-3 which expresses high levels of the protein (Hudziak *et al.*, 1989).

There has been increasing interest in the role of c-*erb*B-2 oncogene in breast cancer, particularly its relationship to prognosis (Barnes, 1989). Overexpression of c-*erb*B-2 oncogene has been shown to correlate with poor prognosis in both primary operable and advanced breast cancer patients by some groups (Varley *et al.*, 1987; Slamon *et al.*, 1987; Walker *et al.*, 1989; Tsuda *et al.*, 1989; Wright *et al.*, 1989; Slamon *et al.*, 1989; Tandon *et al.*, 1989; Paik *et al.*, 1990) but this significant association has not been demonstrated by others (Cline *et al.*, 1987; Van de Vijver *et al.*, 1988*a*; Barnes *et al.*, 1988; Gusterson *et al.*, 1988; Ali *et al.*, 1988; Zhou *et al.*, 1989) and remains controversial. c-erbB-2 oncogene product can be detected immunohistologically in patients with breast cancer. Previous studies with the antibody 21N and others, using southern blotting and immunohistological staining have demonstrated that tumour cell membrane reactivity is related to c-*erb*B-2 gene amplification (Venter *et al.*, 1987; Gusterson *et al.*, 1987). Use of immunohistology to detect elevated levels of c-erbB-2 protein expression allows study of archival tumour samples from well characterised series. In this study we have examined, in a large series of patients managed by a single team, the prognostic effect of c-erbB-2 oncoprotein expression in primary and advanced breast carcinoma and its value in relationship to existing prognostic factors.

### Methods

The patients in this study presented with primary operable or advanced breast cancer to a single surgical team (Professor R.W. Blamey) at the City Hospital, Nottingham. Seven hundred and eighty-two patients with breast cancer were initially entered in the study, 602 consecutive patients with primary operable breast cancer, 57 presenting with stage 3 and 123 with stage 4 disease. Of those patients with primary operable cancer 497 had sufficient tumour material available for immunohistochemical assessment. All 180 cases in the advanced breast cancer group had sufficient histological material, giving a total of 667 suitable cases.

Patients were followed up after surgery at 3 monthly intervals for 18 months and thereafter 6 monthly for 5 years, then annually. Overall survival was taken from the time of original diagnosis to the time of death.

The excised tumours were measured in their fresh state by bisection in two planes, and measurements made in three at right angles. Tumour size was taken as the largest of these three dimensions. The tissue was immersed immediately in

Correspondence: I.O. Ellis, Department of Histopathology, City Hospital, Hucknall Road, Nottingham NG5 1PB, UK.
Received 9 March 1990; and in revised form 29 June 1990.

neutral buffered formalin and allowed to fix for 24 h. The tumour blocks were then processed, embedded routinely in paraffin wax and stored.

A polyclonal antibody 21N (Gullick *et al.*, 1987), raised in rabbits using a synthetic peptide identical in sequence to the predicted C terminus of the c-erbB-2 protein (residues 1243–1255), was used to demonstrate the presence of oncoprotein expression in the primary tumours by a standard immunoperoxidase technique. Three μM sections were cut and dewaxed in xylene and rinsed in absolute alcohol. Endogenous peroxidase activity was blocked with hydrogen peroxide in methanol and non specific binding sites were blocked using 10% normal swine serum. This was followed by incubation in affinity purified primary antibody used at a concentration of 3.93 $\mu g\, ml^{-1}$. This concentration has previously been demonstrated to delineate membrane reactivity in tumours with known oncogene amplification. Binding of the primary antibody was demonstrated by a standard Avidin Biotin Complex technique. This method uses biotinylated swine anti rabbit immunoglobulin followed by preformed soluble complexes of avidin and biotinylated horse radish peroxidase (Dako). The reaction is developed using 0.05% diaminobenzidine with 0.03% hydrogen peroxide in Tris buffer at pH 7.6 for 10 min. The sections were counterstained with haematoxylin. Sections were also processed in the absence of 21N antibody to act as a negative control and two tumours of known immunoreactivity were stained as positive controls.

Studies using the antibody 21N have demonstrated a direct association between tumour cell membrane reactivity and oncogene product expression using western blotting technology. On the basis of these observations tumours were classified according to their immunoreactivity as either positive or negative. Prior to commencement of the study it was decided to assess heterogeneity of immunoreactivity. Only a small proportion of patients showed heterogeneous staining; this was invariably in excess of 50% of tumour cells and subclassification based on this criterion was considered inappropriate. For the purpose of this study these cases exhibiting heterogeneous reactivity were classified as positive. Membrane immunoreactivity was analysed independently by two observers without any prior knowledge of clinical data and in rare cases of discrepancy, a consensus opinion was sought.

In the primary group of patients lymph node sampling was carried out at the time of surgery and on the basis of histological assessment, patients were further categorised into the following groups: lymph node stage A = no nodal involvement, lymph node stage B = low axillary node alone involved, lymph node stage C = apical and/or internal mammary node involved.

Information on histological grade, tumour size, lymph node status, vascular invasion, and survival was recorded for all cases. Oestrogen receptor content was measured at the Tenovus Institute using the Dextran coated charcoal method; a seven point assay was employed and the results were computed by Scatchard analysis. Tumours with an oestrogen receptor content greater than 5 femtomoles per mg of cytosol protein were considered positive. All histological grades were assessed independently by two pathologists (IOE and CWE) using Elston's modification (Elston, 1987) of the Bloom and Richardson method. This technique assesses nuclear pleomorphism, mitotic frequency and tubule formation. Any discrepancy of grading was resolved by review and consensus opinion using a dual headed microscope.

### Results

### *Primary operable breast cancer*

Seventy-five of the 497 patients (15%) of the primary series showed positive membrane immunoreactivity with 21N (Figure 1). Cytoplasmic staining was present to varying degrees but for the purpose of this study was not analysed further. Using life table analysis a highly signficiant correlation was found between poorer survival and invasive tumour cell membrane staining (Figure 2). A significant relationship was also demonstrated between worsening histological grade, which could be assessed in 480 patients, and membrane immunoreactivity (Table I). No correlation was found between membrane expression and lymph node status, oestrogen receptor status and vascular invasion.

**Figure 1** A case of invasive adenocarcinoma of breast showing positive tumour cell membrane immunoreactivity with antibody 21N.

Complete lymph node stage data was available on 479 of these patients. Two hundred and fifty patients were lymph node negative. Comparison of Stage A (lymph node negative) with Stages B and C (operable lymph node positive) patients (Figure 3) according to c-erbB-2 statistics shows no significant difference in survival between c-erbB-2 positive and negative lymph node negative patients. Significant differences in survival were found between c-erbB-2 negative, node negative and positive patients, c-erbB-2 positive node negative and positive patients, and node positive c-erbB-2

**Figure 2** Survival of patients with operable breast carcinoma according to tumour immunoreactivity for 21N.

**Table I** The relationship between histological grade and tumour immunoreactivity for 21N in primary operable breast carcinoma

| | | *21N immunoreactivity* | | |
|---|---|---|---|---|
| | | *Negative* | *Positive %* | *Total* |
| Histological | 1 | 73 | 2 (3) | 75 |
| Grade | 2 | 156 | 19 (11) | 175 |
| | 3 | 181 | 49 (21) | 230 |
| Total | | 410 | 70 (15) | 480 |

$\chi^2 = 18.84$; 2 degrees of freedom $P < 0.0001$.

**Figure 3** Survival for patients with operable breast cancer according to tumour immunoreactivity for 21N and lymph node stage. Pairwise comparison statistics for the four groups gave the following results: a *vs* b $P = 0.23$; a *vs* c $P < 0.0001$; a *vs* d $P < 0.0001$; b *vs* c $P = 0.023$; b *vs* d $P < 0.0001$; c *vs* d $P = 0.003$.

positive and negative patients. A lower prevalance of c-erbB-2 immunoreactivity was observed in node negative disease, 12.4% *vs* stage B/C (node positive disease) – 17.4%.

Multivariate analysis (Cox, 1972) was used to identify whether c-erbB-2 was of independent prognostic significance. In the context of the temporal variables, tumour size and lymph node stage, cell membrane staining was found to have independent significance as a prognostic factor (Table IIIa) but significance was lost when histological grade was included in the analysis (Table IIIb).

### *Advanced breast cancer*

In the group of patients with advanced breast cancer 36 of the 180 patients (20%) showed membrane immunoreactivity. A positive correlation was seen between survival and 21N immunoreactivity in both stage 3 (Figure 4) and stage 4 patients (Figure 5). No association was demonstrated

**Figure 4** Survival of patients with stage 3 breast carcinoma according to tumour immunoreactivity for 21N.

**Figure 5** Survival of patients with stage 4 breast carcinoma according to tumour immunoreactivity for 21N.

between tumour membrane immunoreactivity and tumour size, histological grade, lymph node status and vascular invasion. A weak inverse relationship existed between oestrogen receptor status and oncogene expression; that is tumours showing positive membrane immunoreactivity tended to be oestrogen receptor negative (Table II).

**Table II** The relationship between oestrogen receptor status and tumour immunoreactivity for 21N in patients with advanced breast carcinoma. Oestrogen receptor status was measured on 146 patients

| | | *21N immunoreactivity* | | |
|---|---|---|---|---|
| | | *Negative* | *Positive* | *Total* |
| Oestrogen | Positive | 70 | 10 | 80 |
| Receptor | Negative | 43 | 23 | 66 |
| Status | Total | 103 | 33 | 146 |

$\chi^2 = 9.08$; 1 degree of freedom $P < 0.003$.

**Table III** Results of the Cox Multivariate analysis. 21N immunoreactivity, tumour size and lymph node status were entered with (analysis 1) and without (analysis 2) inclusion of histological grade

| | *Analysis 1* | | *Analysis 2* | |
|---|---|---|---|---|
| | *β coeff.* | *Z value* | *β coeff.* | *Z value* |
| Tumour size | 0.16 | 3.31 | 0.176 | 3.47 |
| Lymph node stage | 0.69 | 7.84 | 0.603 | 6.23 |
| AP21N Immiunoreactivity | Not significant | | 0.528 | 2.85 |
| Histological grade | 0.73 | 6.75 | Not entered | |

Z values of $< 1.96$ are significant ($P < 0.02$).

### Discussion

Assessment of c-*erb*B-2 proto-oncogene overexpression can be achieved using immunocytochemistry on formalin fixed paraffin embedded tumour material to identify membrane localisation of the oncoprotein. Various studies have confirmed a relationship between c-*erb*B-2 gene amplification and immunohistological demonstration of membrane expression of the oncoprotein (Slamon *et al.*, 1987; Venter *et al.*, 1987) and it has been argued that this approach is the most appropriate for routine evaluation (Barnes, 1989). In our series, using the antibody 21N, membrane expression of c-erbB-2 protein was found in 15% of primary carcinomas and in 20% of advanced breast carcinomas. We have demonstrated a statistically significant relationship between poorer survival and positive invasive tumour cell membrane immunoreactivitiy in both primary and advanced breast cancer patients. In primary disease, the relationship was significant only in lymph node positive patients. The lower prevelance of c-erbB-2 positivity in the node negative group may have affected this result. In addition life events occurring in the node negative group are less concentrated in the earlier years of follow-up. We believe that identification of an effect of c-erbB-2 status in node negative patients would require a study of a larger number of patients with longer follow-up. Our series is of particular importance being the largest reported and comprising a consecutive series of patients with primary operable breast cancer presenting to and being treated by a single centre. It should end the controversy concerning the prognostic value of c-erbB-2 immunoreactivity. The findings are consistent with most other reports (Varley *et al.*, 1987; Slamon *et al.*, 1987; Walker *et al.*, 1989; Tsuda *et al.*, 1989; Wright *et al.*, 1989; Slamon *et al.*, 1989; Tandon *et al.*, 1989) but less significant (Van de Vijver *et al.*, 1988*a*; Barnes *et al.*, 1988) and opposing results have been reported (Cline *et al.*, 1987; Gusterson *et al.*, 1988; Ali *et al.*, 1988; Zhou *et al.*, 1989). Although significant, the association shown with survival appears to be less powerful than some existing prognostic factors. The low percentage, 15–20% in most series, of invasive breast carcinoma showing gene amplification requires that large numbers of patients are studied before a significant relationship with prognosis can be demonstrated. This observation alone could explain most of the discrepancies observed between reported series.

Investigation of relationships between c-erbB-2 positive membrane immunoreactivity and established prognostic factors showed no correlation, in our study, with the time dependent variables of lymph node stage and tumour size. This finding is similar to those of some groups (Van der Vijver *et al.*, 1988*a*; Slamon *et al.*, 1987; Tsuda *et al.*, 1989; Tandon *et al.*, 1989; Cox, 1972) but is inconsistent with others (Cline *et al.*, 1987; Rio *et al.*, 1987; Berger *et al.*, 1988; Guerin *et al.*, 1989; Seshadri *et al.*, 1989; Borg *et al.*, 1989) who showed a positive correlation between c-erbB-2 oncoprotein and positive nodal status. Two groups have reported an association with tumour size (Van de Vijver *et al.*, 1988*a*; Borg *et al.*, 1989) but others have not (Cline *et al.*, 1987; Slamon *et al.*, 1987; Tsuda *et al.*, 1989; Wright *et al.*, 1989; Tandon *et al.*, 1989). In the larger series of patients with primary operable cancer we have demonstrated a positive correlation between worsening histological grade and positive membrane immunoreactivity. A similar observation has been made by some groups (Zhou *et al.*, 1987; Barnes *et al.*, 1988; Berger *et al.*, 1988; Walker *et al.*, 1989; Wright *et al.*, 1989; Paik *et al.*, 1990) but others have not identified such a relationship (Rio *et al.*, 1987; Van de Vijver 1988*a*; Guerin *et al.*, 1989). We failed to confirm a similar relationship with histological grade in the advanced breast cancer series. Some of these discrepancies could be explained by differences in selection criteria for patients entered into a particular study and again the low frequency of c-erbB-2 protein expression and low numbers of patients studied.

There are many recognised prognostic factors in human breast cancer. In our breast cancer series we have previously demonstrated that the most powerful factors are lymph node stage, histological grade and tumour size (Todd *et al.*, 1987). The multivariate analysis in this study indicates that c-erbB-2 protein expression is a significant prognostic factor only when assessed with the time related prognostic factors, tumour size and lymph node stage. When the powerful tumour related prognostic factor, histological grade, was introduced into the analysis the independent significance of c-erbB-2 protein expression was lost. c-erbB-2 amplification is found in only a small proportion of tumours and for this reason alone it is perhaps not surprising that it fails to provide prognostic information of a magnitude similar to histological grade. It is difficult to speculate on the potential value of knowledge of elevated c-erbB-2 protein expression without precise knowledge of its function (see below). Speculation that amplification and over expression of certain genes may be reflected in tumour cell morphology (Cardiff, 1988) has been partly borne out by evidence that c-erbB-2 amplification is related to large cell morphology, particularly in ductal carcinoma *in situ* (Van de Vijver *et al.*, 1988*b*). Histological grading is assessed by combining the appearance of various morphological features and mitotic figure frequency (Elston, 1987). It thus provides a summation of a variety of tumour variables. Extrapolating further from the above tentative evidence, one could suggest that histological grade gives an overview of various molecular events affecting morphological appearance. It is unlikely therefore that a single molecular event could compete with histological grade in such a statistical multivariate analysis. We believe the future clinical application of molecular markers of prognosis will be in combination, providing information analogous to histological grade.

Our knowledge of the function of c-erbB-2 oncoprotein is rudimentary. It has similarities to EGFR and there is sufficient evidence to indicate that its role as a membrane receptor for a ligand, yet unknown, is likely. It is persistently overexpressed in a significant proportion of breast carcinomas and clearly delineates a poorer prognostic subgroup. Further support for c-erbB-2's growth regulatory role is the observation that monoclonal antibodies raised against the extracellular domain (Drebin *et al.*, 1986) have exerted an antitumour effect on mutant neu transformed NIH 3T3 cells and on human breast tumour derived cell line. In addition we know that EGFR expression is associated with poorer prognosis and one might postulate that EGFR and c-erbB-2 oncoprotein are both components of a mechanism responsible for breast tumours or progression. Certainly Kadowaki *et al.* (1987) has demonstrated that c-erbB-2 oncoprotein can act as a substrate for EGFR tyrosine kinase. A possible hypothesis, of course, is that binding of ligand to increasing number of receptors leads to an elevation in phosphokinase activity which would promote cell replication. It has recently been demonstrated that a combination of expression of EGFR and c-erbB-2 more efficiently transforms cells than either protein alone (Kokai *et al.*, 1989).

In summary, our study has confirmed that c-erbB-2 overexpression is an important molecular prognostic indicator in breast carcinoma and clearly delineates a poorer prognostic subgroup. This information has clinical implications and if the ligand receptor hypothesis is correct a new chemotherapeutic dimension may be introduced once more knowledge is acquired on a molecular biological level.

We are grateful to Julie Rainbow for preparation of the manuscript and to the Audiovisual Department for preparation of the figures.
This work has been supported by grants from Trent Regional Health Authority, the Cancer Research Campaign and the Imperial Cancer Research Fund.

### References

ALI, I.U., CAMPBELL, G., LIDEREAU, R. & CALLAHAN, R. (1988). Lack of evidence for the prognostic significance of c-*erb*B-2 amplification in human breast carcinoma. *Oncogene Res.*, **3**, 139.

BARGMANN, C.I. & WEINBERG, R.A. (1989). Oncogenic activation of the *neu*-encoded receptor protein by point mutation and deletion. *EMBO J.*, **7**, 2043.

BARNES, D.M. (1988). Editorial. Breast cancer and a proto-oncogene. *Br. Med. J.*, **299**, 1061.

BARNES, D.M., LAMMIE, G.A., MILLIS, R.R., GULLICK, W.J., ALLEN, D.S. & ALTMAN, D.G. (1989). An immunohistochemical evaluation of c-*erb*B-2 expression in human breast carcinoma. *Br. J. Cancer*, **58**, 448.

BERGER, M.S., LOCHER, G.W., SAURER, S. & 4 others (1988). Correlation of the c-*erb*B-2 gene amplification and protein expression in human breast carcinoma with nodal status and nuclear grading. *Cancer Res.*, **48**, 1238.

BORG, A., SIGUARSSON, H., TANDON, A.K. & 4 others (1989). Proto-oncogene amplification in human breast cancer. *Nordic Cancer Union Symposium, Stockholm*, abstract.

CARDIFF, R.D. (1988). Cellular and molecular aspects of neoplastic progression in the mammary gland. *Eur. J. Cancer Clin. Oncol.*, **24**, 15.

CLINE, M.J., BATTIFORA, H. & YOKOTA, J. (1987). Proto-oncogene abnormalities in human breast cancer: correlations with anatomic features and clinical course of disease. *J. Clin. Oncol.*, **5**, 999.

COUSSENS, L., YANG-FENG, T.L., LIAO, Y.-C. & 9 others (1985). Tyrosine kinase receptor with extensive homology to EFG receptor shares chromosomal location with *neu* oncogene. *Science*, **230**, 1132.

COX, D.R. (1972). Regression models and life tables. *J. Roy. Statist. Soc.*, **B**, 341.

DI FIORE, P.P., PIERCE, J.H., KRAUS, M.H., SEGATTO, O., KING, C.R. & AARANSON, S.A. (1987). c-*erb*B-2 is a potent oncogene when overexpressed in NIH/3T3 cells. *Science*, **237**, 178.

DREBIN, J.A., LINK, V.C., WEINBERG, R.A. & GREENE, M.I. (1986). Inhibition of tumour growth by a monoclonal antibody reactive with an oncogene encoded tumur antigen. *Proc. Natl Acad. Sci. USA*, **83**, 9129.

ELSTON, C.W. (1987). Grading of invasive carcinomas of the breast. In: *Diagnostic Histopathology of the Breast* Page, D.L. & Anderson, T.J. (eds). Churchill Livingstone: Edinburgh, 300.

GUERIN, M., GABILLOT, M., MATHIEU, M.-C. & 4 others (1989). Structure and expression of c-*erb*B-2 and EGF receptor genes in inflammatory and non-inflammatory breast cancer: prognostic significance. *Int. J. Cancer*, **43**, 201.

GULLICK, W.J., BERGER, M.S., BENNETT, P.L.P., ROTHBARD, J.B. & WATERFIELD, M.D. (1987). Expression of the c-erbB-2 protein in normal and transformed cells. *Int. J. Cancer*, **40**, 7935.

GULLICK, M.W. & VENTER, D.J. (1989). The c-*erb*B-2 gene and its expression in human tumours. *The Molecular Biology of Cancer.* Waxman, J. & Sikora, K. (eds). Blackwells: Oxford, UK, 38.

GUSTERSON, B.A., GULLICK, W.J., VENTER, D.J. & 5 others (1987). Immunohistochemical localisation of c-erbB-2 in human breast carcinomas. *Molecular and Cellular Probes*. **1**, 383.

GUSTERSON, B.A., MACHIN, L.G., GULLICK, W.J. & 6 others (1988). c-*erb*B-2 expression in benign and malignant breast disease. *Br. J. Cancer*, **5**, 453.

HUDZIAK, R.M., LEWIS, G.-D., WINGET, M., FENDLY, B.M., SHEPARD, H.M. & ULLRICH, A. (1989). p185$^{HER2}$ monoclonal antibody has antiproliferative effects *in vitro* and sensitised human breast tumour cells to tumour necrosis factor. *Mol. Cell Biol.*, **9**, 1165.

HUDZIAK, R.M., SCHLESSINGER, J. & ULLRICH, A. (1987). Increased expression of the putative growth factor receptor p185$^{HER2}$ causes transformation and tumour genesis of NIH 3T3 cells. *Proc. Natl Acad. Sci. USA*, **84**, 7159.

KADOWAKI, T., KASUGA, M., TOBE, K. & 7 others (1987). A Mr = 190,000 glycoprotein phosphorylated on tyrosine residues in Epidermal Growth Factor Receptor stimulated KB cells is the product of c-erbB-2 gene. *Biochem. Biophys. Res. Com.*, 144.

KOKAI, Y., MYERS, J., WADA, T. & 5 others (1989). Synergistic interaction of p185c neu and the EFG receptor leads to transformation of rodent fibroblasts. *Cell*, **58**, 287.

LAMMIE, G.A., BARNES, D.M., MILLIS, R.R. & GULLICK, W.J. (1989). An immunohistochemical study of the presence of c-erbB-2 protein in Paget's disease of the nipple. *Histopathology in Press*, 1989.

MAGUIRE, H.C. & GREENE, M.I. (1989). The *neu* (c-*erb*B-2) oncogene. *Seminars in Oncol.*, **16**, 148.

PAIK, S., HUZAN, R., FISHER, E.R. & 6 others (1990). Pathological findings from the National Surgical Adjuvant Breast and Bowel Project: Prognostic Significance of erbB-2 Protein over-expression in primary Breast Cancer. *J. Clin. Oncol.*, **8**, 103.

RIO, M.C., BELLOCQ, J.P., GAIRARD, B. & 7 others (1987). Specific expression of the pS2 gene in subclasses of breast cancers in comparison with expression of the oestrogen and progesterone receptors and the oncogene. *erb*B2. *Proc. Natl Acad. Sci. USA*, **84**, 9243.

SESHADRI, R., MATTHEWS, C., DOBROVIC, A. & HORSFALL, D.J. (1989). The significance of oncogene amplification in primary breast cancer. *Int. J. Cancer*, **43**, 270.

SLAMON, D.J., CLARK, G.M., WONG, S.G., LEVIN, W.J., ULLRICH, A. & MCGUIRE, W.L. (1987). Human breast cancer: Correlation of relapse and survival with amplification of the *HER-2/Neu* oncogene. *Science*, **235**, 177.

SLAMON, D.J., GOLDOLPHIN, W., JONES HOLD, J.A. & 7 others (1989). Studies of HER-2/neu proto-oncogene in human breast and ovarian cancer. *Science*, **244**, 707.

STERNBERG, M.J.E. & GULLICK, W.J. (1989). Neu receptor dimerisation. *Nature*, **339**, 587.

TANDON, A.K., CLARK, G.M., CHAMNESS, G.C., ULLRICH, A. & MCGUIRE, W.L. (1989). Her-2/neu oncogene protein and prognosis in breast cancer. *J. Clin. Invest.*, **7**, 1120.

TODD, J.H., DOWLE, C., WILLIAMS, M.R. & 5 others (1987). Confirmation of a prognostic index in primary breast cancer. *Br. J. Cancer*, **56**, 489.

TSUDA, H., HIROHASHI, S., SHIMOSATO, Y. & 11 others (1989). Correlation between long term survival in breast cancer patients and amplification of two putative oncogene-coamplification units: hst-1/int-2 and c-erbB-2/ear-1. *Cancer Res.*, **49**, 3104.

VAN DE VIJVER, M.J., MOOI, W.J., PETERSE, J.L. & NUSSE, J.L. (1988*a*). Amplification and overexpression of the new oncogene in human breast carcinomas. *Eur. J. Surg. Oncol.*, **14**, 111.

VAN DE VIJVER, M.J., PETERSE, J.L., MOOI, W. & 4 others (1988*b*). Neu-protein overexpression in breast cancer. Association with comedo-type ductal carcinoma in situ and limited prognostic value in stage II breast cancer. *N. Engl. J. Med.*, **319**, 1239.

VARLEY, J.M., SWALLOW, J.E., BRAMMAR, W.J., WHITTAKER, J.L. & WALKER, R.A. (1987). Alterations to either c-*erb*B-2 (*neu*) or c-*myc* proto-oncogenes in breast carcinomas correlate with poor short term prognosis. *Oncogene*, **1**, 423.

VENTER, D.J., KUMAR, S., TUZI, N. & GULLICK, W.J. (1987). Overexpression of the c-erbB-2 oncoprotein in human breast carcinomas: immunohistochemical assessment correlated with gene amplification. *Lancet*, **ii**, 69.

WALKER, R.A., GULLICK, W.J. & VARLEY, J.M. (1989). An evaluation of immunoreactivity for c-erbB-2 protein as a marker of poor short-term prognosis in breast cancer. *Br. J. Cancer*, **60**, 426.

WEINER, D.B., LIU, J., COHEN, J.A., WILLIAMS, W.V. & GREENE, M.I. (1989). A point mutation in the *neu* oncogene mimics ligand induction of receptor aggregation. *Nature*, **339**, 230.

WRIGHT, C., ANGUS, B., NICHOLSON, S. & 6 others (1989). Expression of c-erbB-2 oncoprotein: a prognostic marker in human breast cancer. *Cancer Res.*, **49**, 2087.

YARDEN, Y. & WEINBERG, R.A. (1989). Experimental approaches to hypothetical hormones: detection of a candidate ligand of the *neu* proto-oncogene. *Proc. Natl Acad. Sci. USA*, **86**, 3179.

ZHOU, D.-J., AHUJA, H. & CLINE, M.J. (1989). Proto-oncogene abnormalities in human breast cancer: c-*erb*B-2 amplifcation does not correlate with recurrence of disease. *Oncogene*, **4**, 105.

ZHOU, D., BATTIFORA, H., YOKOTA, J., YAMAMOTO, T. & CLINE, M.J. (1987). Association of multiple copies of the c-*erb*B-2 oncogene with spread of breast cancer. *Cancer Res.*, **47**, 6123.