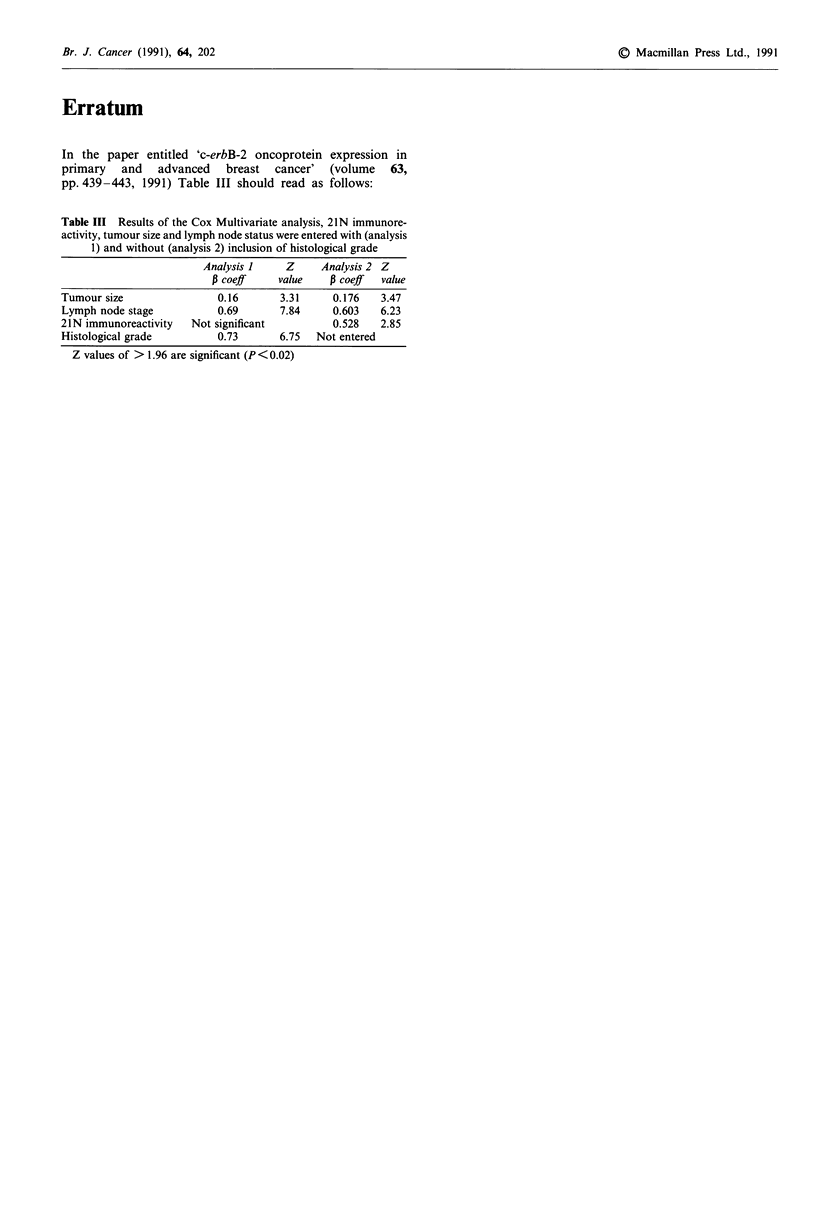# Erratum

**Published:** 1991-07

**Authors:** 


*Br. J. Cancer* (1991), **64**, 202 © Macmillan Press Ltd., 1991

### Erratum

In the paper entitled 'c-*erb*B-2 oncoprotein expression in primary and advanced breast cancer' (volume **63**, pp. 439–443, 1991) Table III should read as follows:

**Table III** Results of the Cox Multivariate analysis, 21N immunoreactivity, tumour size and lymph node status were entered with (analysis 1) and without (analysis 2) inclusion of histological grade

| | *Analysis 1 β coeff* | *Z value* | *Analysis 2 β coeff* | *Z value* |
|---|---|---|---|---|
| Tumour size | 0.16 | 3.31 | 0.176 | 3.47 |
| Lymph node stage | 0.69 | 7.84 | 0.603 | 6.23 |
| 21N immunoreactivity | Not significant | | 0.528 | 2.85 |
| Histological grade | 0.73 | 6.75 | Not entered | |

Z values of $>1.96$ are significant ($P<0.02$)